# Development of a Tailored Mobile Phone–Based Intervention to Facilitate Parent-Child Communication and Build Human Papillomavirus Vaccine Confidence: Formative Qualitative Study

**DOI:** 10.2196/43041

**Published:** 2023-04-04

**Authors:** Jennifer Cunningham-Erves, Consuelo H Wilkins, Amanda F Dempsey, Jessica L Jones, Chris Thompson, Kathryn Edwards, Megan Davis, Lindsay S Mayberry, Douglas Landsittal, Pamela C Hull

**Affiliations:** 1 Department of Internal Medicine Meharry Medical College Nashville, TN United States; 2 Vanderbilt University Medical Center Nashville, TN United States; 3 Merck Denver, CO United States; 4 Meharry Vanderbilt Alliance Nashville, TN United States; 5 233 Analytics Nashville, TN United States; 6 Indiana University Bloomington, IN United States; 7 University of Kentucky Lexington, KY United States

**Keywords:** human papillomavirus, HPV, vaccine, hesitancy, parent-child communication, theory, mobile health, mHealth, adolescents, patient education

## Abstract

**Background:**

Human papillomavirus (HPV) vaccine hesitancy is on the rise, and provider communication is a first-line strategy to address parental concerns. The use of the presumptive approach and motivational interviewing by providers may not be enough to influence parental decision-making owing to the providers’ limited time, self-efficacy, and skills to implement these strategies. Interventions to enhance provider communication and build parental HPV vaccine confidence have been undertested. Delivering tailored patient education to parents via mobile phones before they visit the health care provider may address time constraints during clinic visits and positively affect vaccine uptake.

**Objective:**

This study aimed to describe the development and evaluate the acceptability of a mobile phone–based, family-focused intervention guided by theory to address concerns of HPV vaccine–hesitant parents before the clinic visit, as well as explore intervention use to facilitate parent-child communication.

**Methods:**

The health belief model and theory of reasoned action guided intervention content development. A multilevel stakeholder engagement process was used to iteratively develop the *HPVVaxFacts* intervention, including a community advisory board review, a review by an advisory panel comprising HPV vaccine–hesitant parents, a health communications expert review, semistructured qualitative interviews with HPV vaccine–hesitant parents (n=31) and providers (n=15), and a content expert review. Inductive thematic analysis was used to identify themes in the interview data.

**Results:**

The qualitative interviews yielded 4 themes: overall views toward mobile device use for health information, acceptability of *HPVVaxFacts*, facilitators of *HPVVaxFacts* use, and barriers to *HPVVaxFacts* use. In parent interviews after reviewing *HPVVaxFacts* prototypes, almost all parents (29/31, 94%) stated they intended to have their child vaccinated. Most of the parents stated that they liked the added *adolescents’ corner* to engage in optional parent-child communication (ie, choice to share and discuss information with their child; 27/31, 87%) and shared decision-making in some cases (8/31, 26%). After incorporating all input, the final intervention consisted of a 10-item survey to identify the top 3 concerns of parents, followed by tailored education that was mapped to each of the following concerns: evidential messages, images or graphics to enhance comprehension and address low literacy, links to credible websites, a provider video, suggested questions to ask their child’s physician, and an optional adolescents’ corner to educate the patient and support parent-child communication.

**Conclusions:**

The multilevel stakeholder-engaged process used to iteratively develop this novel intervention for HPV vaccine–hesitant families can be used as a model to develop future mobile health interventions. This intervention is currently being pilot-tested in preparation for a randomized controlled trial aiming to increase HPV vaccination among adolescent children of vaccine-hesitant parents in a clinic setting. Future research can adapt *HPVVaxFacts* for other vaccines and use in other settings (eg, health departments and pharmacies).

## Introduction

### Background

Improving human papillomavirus (HPV) vaccination rates among adolescents is a public health priority [1]. In the United States, the HPV vaccine completion rate among adolescents aged 13 to 17 years was 61.7% in 2021 [2], well below the national goal of 80% [3]. This leaves many adolescents at risk for preventable HPV infection and associated sequelae [4-6]. HPV vaccination rates declined owing to the COVID-19 pandemic, with a drop of up to 77% in April 2020 compared with prior years [7,8]. Parental vaccine hesitancy is a major contributor to suboptimal HPV vaccination rates [9,10], and a recent study suggests that it may be becoming more common. From 2012 to 2018, the proportion of unvaccinated adolescents receiving a provider recommendation for HPV vaccine increased from 27% to 49.3%; yet, the lack of intent to have their child vaccinated increased among parents with boys (from 44.4% to 59.2%) as well as among those with girls (from 54.1% to 68.1%) [11].

A strong recommendation and effective communication from a provider are critical to address parental vaccine hesitancy and increase uptake [12-14]. Children are almost 10 times more likely to receive the HPV vaccine if their parents receive a strong recommendation [15]. Motivational interviewing to guide conversations with vaccine-hesitant parents during a clinic visit has also been impactful [16]. This is critical because one-quarter of parents report secondary acceptance (ie, a parent who declines the HPV vaccine and later accepts it) after learning about the vaccine and the diseases it prevents [17]. Nevertheless, providers often have difficulty conversing with vaccine-hesitant parents and patients, and time constraints limit clinic visit discussion or communication training participation. Some providers also have low self-efficacy and limited skills to discuss the vaccine [16,18-21]. Therefore, many do not provide a recommendation and communication or give a poor-quality recommendation and communication [15]. This has led to some parents becoming dissatisfied with patient-provider communication, undermining their understanding and confidence in the HPV vaccine [22-25]. This further limits many parents’ ability to discuss and make HPV vaccination decisions with their adolescents [26].

Communication can come in several forms from providers. Nevertheless, few research studies explore modalities used by providers or parent and patient preference of these modalities [22]. Technology is a form of communication that can be used to provide information outside of the clinic visit [27] and address some provider barriers. Mobile health (mHealth) interventions are innovative and promising strategies to positively affect beliefs and increase knowledge, intent, and HPV vaccination [28,29]. A handful of mobile phone apps for the HPV vaccine have been found acceptable and useful by parents [30-33]. To our knowledge, only one of these has been tested for efficacy, and it improved parental attitudes and increased HPV vaccine initiation and completion rates among adolescent girls [33]. However, these mHealth interventions focus on general HPV vaccine promotion and not the unique needs or concerns of HPV vaccine–hesitant parents and adolescents. In addition, they were designed to focus on younger adolescents or girls only, or they included limited testing across minoritized groups.

Offering previsit patient education could be an effective complement to provider communication strategies offered during a clinic visit to promote uptake among HPV vaccine–hesitant parents [22]. Previous studies have found knowledge to be a premotivational factor to influence attitudes and intentions [34], which are major drivers in vaccine uptake when the opportunity is available [35,36]. The use of individually tailored messages could be effective [37] to address the unique educational needs of vaccine-hesitant parents after the initial presumptive recommendation fails [38]. Similar to past studies [39-43], our preliminary data suggest that parents want HPV vaccine information from a provider *before* a clinic visit to have time to process the information and, in some cases, engage in communication and shared decision-making with their child (Cunningham-Erves, J, unpublished data, June 2022).

### Objectives

Our primary goal was to develop an individually tailored educational intervention for HPV vaccine–hesitant parents that providers could deliver before clinic visits to increase HPV vaccine confidence and uptake. The secondary goals were for this intervention to facilitate parent-child communication, reduce parent-child anxiety, and minimize provider time burden during clinic visits. We explored (1) intervention acceptability from HPV vaccine–hesitant parents undecided about the HPV vaccine and health care providers serving adolescents and (2) potential use of this intervention to facilitate parent-child communication.

## Methods

### Overview

**Figure 1:**
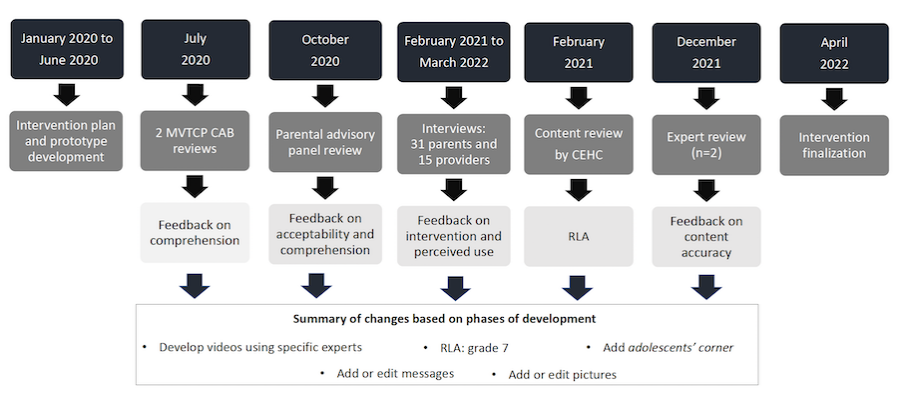
Mobile phone–based intervention HPVVaxFacts development process. CAB: community advisory board; CEHC: Center for Effective Health Communication; MVTCP: Meharry-Vanderbilt–Tennessee State University Cancer Partnership; RLA: reading-level assessment.

We conducted a formative research study, applying community-engaged research principles to develop *HPVVaxFacts*, a mobile phone–based intervention aimed to increase HPV vaccine confidence among vaccine-hesitant parents and decisions to have their children vaccinated. A 7-phase process was used for intervention development. Figure 1 depicts the phases and timeline for intervention development.

### Ethics Approval, Informed Consent, and Participation

This research was approved by the institutional review board of Meharry Medical College (18-12-890). Written consent was obtained from parents and providers before their interviews. Interview transcriptions were deidentified and assigned a code before data analysis to protect the participants’ confidentiality. Providers were compensated with a US $50 gift card, and parents were compensated with a US $40 gift card.

### Phase 1: Intervention Development (January 2020 to June 2020)

#### Overview

Our research team developed the initial intervention plan, content, and prototype for the mobile phone–based intervention, *HPVVaxFacts*, drawing on the skills, expertise, and experiences of our team*.* This intervention aimed to increase HPV vaccine uptake among vaccine-hesitant parents. The transdisciplinary team included community engagement specialists; an immunization delivery specialist; behavioral scientists; a vaccinology expert; and technology professionals from a software development company, 233 Analytics LLC, based in Nashville, Tennessee, United States. To inform the initial intervention development plan, we conducted a literature review to identify parental HPV vaccine concerns and existing interventions that address these concerns. Example interventions that influenced our work are *Teen VaxScene* [44] and CHICOs [45].

#### Theoretical Application

The theory of reasoned action (TRA) [46] and health belief model (HBM) [47] were used to guide intervention development. According to the TRA, child HPV vaccination would more likely occur if a parent has high or positive intentions. Parental intentions are influenced by their attitudes toward the vaccine and their social normative perceptions of their child being vaccinated. Distal variables (eg, knowledge and sociodemographics) could indirectly influence attitudes and social norms, which subsequently affect intention [46]. HBM constructs were applied to explore the attitude component of the TRA. According to the HBM, parents will have higher vaccination intentions if they perceive that the child is at risk for HPV, HPV sequalae are severe (eg, cancers of the cervix or head and neck), and the benefits of HPV vaccination outweigh perceived barriers and risks of vaccination [47].

#### Intervention Components and Delivery

*HPVVaxFacts* provides individually tailored education to vaccine-hesitant parents and is intended to be delivered to the parents’ mobile phone via a link in an SMS text message up to 2 weeks before an upcoming clinic visit. To tailor the education, a parent answers a 10-item survey adapted from the Vaccination Confidence Scale [48] to identify their top 3 concerns about the HPV vaccine. Examples of the survey items are “I wonder if getting many different vaccines could cause harm to my child’s health” and “I wonder if natural immunity is better than getting the HPV vaccine.” Parents select their level of agreement with each item. The concerns are then ranked using the survey responses, with higher scores indicating higher levels of vaccine hesitancy. If a parent records the same response across >1 question, the algorithm ranks the concerns using a default order established by the team based on existing literature. Next, the top 3 concerns are provided to the parent, and the parent is allowed to change the ranking if they disagree with the order of concerns. Parents then review the tailored content associated with each of the top concerns, which includes concern-specific images or graphics, links to other reliable websites, a video of a provider discussing the concern, and suggested questions they can ask their child’s physician to promote shared decision-making.

Each parental concern also has an optional button called *Share with your kids*. Each child’s tailored page begins with a question about the concern and then provides 3 to 4 short bullet points to answer the question. Example questions include “Why do I need more shots?” and “Are the HPV shots needed to protect my health or can my body protect itself from HPV?” A picture is provided for each concern to enhance comprehension. The content’s source is provided at the end of the information. Parents can determine which, if any, concerns they are comfortable sharing and discussing with their child.

Upon completion, the parent is prompted to save the information about their top concerns and send it to themselves via SMS text message or email or print it out for future reference and to discuss the concerns with their provider at the upcoming visit. A *Contact Us* tab is also available on the web page to enable parents to contact the researchers to answer any additional questions. Parents are sent a reminder if they complete the 10-item survey but do not review the information content. It takes parents 7 to 10 minutes to navigate and review the site.

### Phase 2: Meharry-Vanderbilt–Tennessee State University Cancer Partnership Cancer Outreach Core Community Advisory Board Review (July 2020)

Established in 2011, the Meharry-Vanderbilt–Tennessee State University Cancer Partnership (MVTCP) Cancer Outreach Core community advisory board (CAB) is an academic-community partnership that provides consultation on numerous MVTCP research projects and supports implementation of clinical trials. The CAB meets quarterly and includes >20 community members from diverse racial and ethnic backgrounds who are cancer survivors, caregivers, representatives of cancer-related organizations, and other community members with an interest in cancer prevention and control [49]. Members of the CAB were invited to review and provide feedback on, and strategies to improve, the *HPVVaxFacts* intervention to ensure acceptability and appropriateness. The research team incorporated the feedback into the intervention design and content.

### Phase 3: Parental Advisory Panel Review (October 2020)

We also created an advisory panel of 5 African American mothers who were hesitant to get their child the HPV vaccine. They were invited from an existing database of past research participants who agreed to be contacted for future studies. We presented the mobile phone–based intervention and message content to the mothers, and they provided feedback on the extensiveness, comprehension, and cultural appropriateness of the design and content. The research team discussed the feedback and revised the intervention accordingly. This one-time review occurred via a 90-minute Zoom (Zoom Video Communications, Inc) session, with US $20 paid as compensation to the mothers.

### Phase 4: Center for Effective Health Communication Content Review (February 2021)

The Center for Effective Health Communication (CEHC) located at Vanderbilt University promotes improved health care quality and outcomes via effective health information exchange. For our study, the CEHC consultants included a behavioral scientist, a physician, and a medical sociologist. They reviewed the content to ensure that it would be comprehensible to parents and their adolescent children with varying literacy skills and diverse cultures. Specifically, they proposed content changes to reflect best practices for effective communication (eg, communicating 1 thought per sentence, simplifying sentence structure, and removing or clearly defining medical jargon) along with pre-post information on readability (ie, Flesch-Kincaid Grade Level, Flesch Reading Ease, and words per sentence) [50].

### Phase 5: Semistructured Interviews With Parents and Providers (February 2021 to June 2021)

#### Study Design

We used a qualitative, descriptive study design to understand decision-making on the HPV vaccine and gather feedback on the intervention among parents who self-identified as vaccine hesitant and among providers who served adolescent children of vaccine-hesitant parents. We conducted semistructured interviews and then iteratively revised the intervention design and content (ie, quiz items, draft messages, images or graphics, and layout) using interview data to guide optimization.

#### Sampling and Recruitment

Parents were recruited from the southeastern region of the United States. We recruited a purposeful criterion sample, which is a selection of individuals who are especially knowledgeable about, or have experience regarding, a phenomenon of interest [51,52]. Parents were eligible if they had children aged 11 to 18 years who were unvaccinated against HPV, were undecided or had delayed or refused the HPV vaccine in the past 2 years, had access to digital technology, and spoke English. They were identified using ResearchMatch, a web-based registry of research study volunteers [53]. Providers were identified using purposeful snowball sampling. This means participating providers referred other providers from their networks throughout the United States [51,52]. Providers were eligible if they were physicians, physician assistants, or nurse practitioners who deliver primary care to adolescent patients aged 11 to 18 years. Providers were contacted via mail, email, or word of mouth.

#### Data Collection and Analysis

We drafted an open-ended interview protocol for parents and another one for providers to elicit (1) attitudes about the HPV vaccine and actual or perceived facilitators and barriers to HPV vaccination and (2) feedback on the wording, aesthetics, and format of draft intervention content and delivery to inform revisions. Data collection for parents occurred from February 2021 to May 2021 and for providers from May 2021 to March 2022. Before the interview, parents were sent a link via email for the informed consent document and an adapted 10-item survey to be used in the intervention. The survey assessed parental concerns about the HPV vaccine from the parent and provider perspectives. These data were collected via REDCap (Research Electronic Data Capture; Vanderbilt University), a secure web-based data collection application [54]. On the day of the interview, parents were reminded of the study’s purpose. Interviews lasted 30 to 45 minutes for providers and 45 to 60 minutes for parents. The lead author (JC-E) and a graduate student (MD), both trained in qualitative research methodology, administered the survey and conducted the interviews.

#### Analytic Plan

SPSS software (version 28.0; IBM Corp) was used to analyze survey data. Descriptive analyses (eg, means and frequencies) were used to describe patterns in the data from the surveys of parents and providers. Qualitative data analysis was conducted by the lead author (JC-E) and a graduate student (MD), both trained in qualitative research methods. An a priori hierarchical coding system was developed based on the interview protocol and a preliminary review of the transcripts. Reliability was established in the coding system, followed by independent reviews of each transcript. Next, codes were compared and discrepancies resolved. Using an iterative inductive-deductive approach, codes were merged to form higher-order themes. Strategies to ensure rigor included triangulation, thick descriptions, and peer debriefing [55].

### Phase 6: Expert Review (December 2021)

Two experts conducted a final review of the message library content for accuracy. The content reviewers were an expert in vaccinology (including clinical trials, vaccines, and infectious diseases such as HPV; KE) and an expert in pediatrics and immunization delivery (including HPV vaccination; AFD). Content was deemed evidential if the experts did not find inaccurate or obsolete information using existing peer-reviewed literature. Corrections were completed if identified by the experts.

### Phase 7: Intervention Finalization (February 2022)

The research team made a final round of modifications to the intervention to ensure incorporation of all the key interview findings of parents and providers and the feedback from the CAB members, parental advisory panel, and vaccine experts. Areas of refinement included the individually tailored message concepts, images, and design of the intervention. The review by HPV vaccine experts and CEHC consultants ensured that the content in the messaging library was accurate and comprehensible. Finally, we confirmed that the tailoring variables were matched to specific educational messages identified by parents.

## Results

### Phase 2: MVTCP CAB Review

Overall, the MVTCP CAB members acknowledged that addressing HPV vaccination among adolescents was an important strategy to reduce cancer disparities. They perceived that the intervention was necessary, “eye-catchy,” and user-friendly and addressed specific parental concerns. However, they stated that more diversity in sexual orientation as well as race and ethnicity was needed in the pictures presented on the home page to promote inclusiveness. In addition, they suggested that the intervention should include more visuals to reflect HPV vaccination for male patients. Finally, participants suggested lowering the literacy level because the terms seemed to be “too scientific” or “high grade level.”

### Phase 3: Parental Advisory Panel Review

The top concern among the parents on the advisory panel was the short- and long-term side effects of the vaccine. They also questioned whether and why the vaccine was needed at the recommended age, which they perceived to be young. The parents demonstrated overall enthusiasm with the mobile phone–based intervention and perceived that it increased their knowledge of HPV and the vaccine. They particularly liked the colors and the “clean look” achieved via the use of white space throughout the intervention. The question-and-answer choices were approved, and a suggestion was made to add the option for parents to modify the ranking of top concerns that was generated based on their survey responses. The format to provide content was deemed acceptable. However, the parents did not perceive that the home page depicted enough diversity. They suggested adding an African American family, particularly a man and son, with the other parents. Another suggestion was to add pictures, including those of (1) men throughout the intervention because it appeared too female oriented and (2) medical doctors who reflect “all races in white coats.” The parents preferred a brief video for each vaccine with comprehensible and accurate content. The final suggestion was to be “kid friendly.” The parents requested the addition of an *adolescents’ corner* (ie, images, messages, and testimonials designed for children) to enhance discussion and potential shared decision-making between the parent and the adolescent regarding the HPV vaccine.

### Phase 4: Feedback From CEHC

The Flesch-Kincaid Grade Level was 8.0 before the CEHC review compared with 7.2 after the CEHC review. The Flesch Reading Ease score was 65.6 before the CEHC review and 68.2 after the CEHC review. Finally, there were 16 words per sentence before the CEHC review and 14 words per sentence after the CEHC review. This was better than the recommended range of 15 to 20 words per sentence. Additional recommendations were related to grammar (eg, reordering or removal of information and removing potentially manipulative or contradictory language).

### Phase 5: Semistructured Interviews With Parents and Providers

#### Sociodemographics

Of the 31 parents, 18 (58%) were White, 25 (81%) were women, and 19 (61%) had a bachelor’s degree or higher. Although there was a wide range in terms of income, 55% (16/29) had an annual household income of <US $80,000. Of the 15 providers, 10 (67%) were White, 11 (74%) were women, 13 (87%) were pediatricians, and 10 (67%) practiced in an urban area (Table 1).

**Table 1:** Sociodemographics of parents (n=31) and providers (n=15).

Characteristics				Values				
**Parents (n=31), n (%)**								
	**Race**							
		African American					12 (39)	
		White					18 (58)	
		Latinx					1 (3)	
	**Sex**							
			Male		6 (19)			
			Female		25 (81)			
	**Education level**							
		GED^a^ or high school diploma				2 (7)		
		Associate degree				5 (16)		
		Some college				5 (16)		
		Bachelor’s degree				10 (32)		
		Postgraduate degree				9 (29)		
	**Annual household income (US $)**							
		<20,000					3 (10)	
		20,001 to 40,000					4 (13)	
		40,001 to 60,000					5 (16)	
		60,001 to 80,000					4 (13)	
		>80,000					13 (42)	
		Did not want to answer					2 (7)	
**Providers (n=15)**								
	**Race, n (%)**							
		African American					2 (13)	
		White					10 (67)	
		Other					3 (20)	
	**Sex, n (%)**							
		Male						4 (27)
		Female						11 (73)
	**Practice type, n (%)**							
		Pediatrics					13 (87)	
		Family medicine					1 (7)	
		Adolescent medicine					1 (7)	
	**Practice area, n (%)**							
		Urban						10 (67)
		Suburban						4 (27)
		Rural or mostly mural						1 (7)
	Years in practice, mean (SD)						14.3 (8.6)	
	Residency year training (range)						1982-2021	

^a^GED: General Educational Development Test.

#### Findings From Semistructured Interviews

We identified 5 themes, and each is outlined in the following sections.

##### Theme 1: Overall Views Toward Mobile Device Use for Health Information

Almost all parents and providers stated that mobile devices were acceptable for receiving health information related to their children. They preferred that the medical home deliver the health information to the parents’ mobile device to ensure accuracy and increase the likelihood of trusting the information for use in decision-making. Each unique perspective on overall mobile device use for health information has been provided in the paragraphs that follow.

Nearly all parents reported that they knew how to find health information on apps. The use of mobile web pages was common, easy, and convenient. The types of health information previously accessed via mobile device included laboratory reports after a health care visit and Google to access health information sites. *Reputable sources* included MyChart, WebMD, the Centers for Disease Control and Prevention, scientific studies, and university- and hospital-sponsored websites. The reasons for a small number of parents not using mobile devices included the inability to identify credible sources, perceived lack of privacy and confidentiality, and difficulty understanding web-based information. However, all parents used their mobile phone daily to access information in general.

Most of the providers stated that there is an increasing trend in mobile device use to access health information, and nearly all perceived that this was due to societal norms. The use of mobile devices was also perceived to be driven by medical homes using telemedicine and web-based health portals to engage patients. As a result, most of the providers believed that accessing a mobile phone–based intervention would be easy and convenient for most parents.

##### Theme 2: Acceptability of HPVVaxFacts (Parents Only)

All participants found the intervention acceptable. The layout, including the survey (*quiz*), was viewed as “nice and simple,” “quick,” and “easy to navigate.” The receipt of content tailored to parents’ top 3 concerns was appreciated. The revised visuals were generally considered relatable and diverse in race. However, the visuals were still perceived as female oriented, suggesting an ongoing need for balance by sex. A few of the parents further suggested the addition of credible sources at the end of content for each concern. Minor suggestions included increasing font size and editing directions for site navigation for clarity. For messages specifically, parents accepted the content with suggestions to improve clarity and reduce the length on specific concerns. Details on message content and its development will be provided in a separate paper. The iterative changes led to few or no suggestions for improvement near the end of the interviews. At the postintervention review, almost all parents (29/31, 94%) stated that they intended to have their child vaccinated. Most of the parents stated that they liked the added *adolescents’ corner* to engage in optional parent-child communication (ie, choice to share and discuss information with their child; 27/31, 87%) and shared decision-making in some cases (8/31, 26%).

##### Theme 3: Facilitators of HPVVaxFacts Use

Both parents and providers stated that intervention delivery via mobile phone would be easy and convenient based on prior experiences. This allows time to review messages before the clinic visit. However, they differed in the timing of intervention delivery. All parents wanted information from 2 weeks to a month before the clinic visit. This would allow them to process the information and have conversations with their family. The providers recognized clinic-specific time constraints and how this intervention would be beneficial before the clinic visit. However, a small number of providers perceived that providing information weeks to months in advance would be too far ahead, and, instead, they thought that delivery in the waiting room immediately before the clinic visit would be ideal. Each unique perspective on facilitators of *HPVVaxFacts Use* is provided in the paragraphs that follow.

Overall, parents reported that they would use the mobile phone–based intervention before a clinic visit. They perceived that the previsit timing gives the parent control and empowerment in the decision-making process. It prevents the parent from feeling “pressured” to make a quick decision in the physician’s office. In addition, many parents stated that the previsit information would allow them time to think about and discuss the vaccine with their family, spouse, and children. Most of the parents liked the fact that *HPVVaxFacts* would come from their child’s physician, a trusted health information source. Finally, parents perceived the use of mobile devices to access this intervention as a social norm.

Most of the providers believed that the *HPVVaxFacts* intervention would increase parental engagement with them in the HPV vaccine decision-making process. They stated that delivery via mobile phone increases the likelihood of parental acceptance of this intervention. Many of the providers perceived that the delivery of the intervention before the visit would improve, and reduce the time needed for, patient-provider communication. Vaccine-hesitant parents would receive information that would be tailored to their needs or allow them to better understand the questions they have on the HPV vaccine before talking with the provider. Furthermore, through understanding the intervention content, providers could better prepare to answer parents’ specific questions about HPV or the vaccine.

##### Theme 4: Barriers to HPVVaxFacts Use

Parents and providers identified potential barriers to using *HPVVaxFacts* that were distinct by group.

A commonly cited barrier was concern about a breach of privacy and confidentiality. A parent feared being exposed to a security breach because data would be stored in the “cloud.” Therefore, this parent stated that they were more likely to use the intervention through a health portal than through a mobile app. A parent wanted additional guidance on intervention navigation. Some of the parents cited a possible lack of accessibility for other parents who do not have a computer or lack access to a mobile phone. Suggestions to increase access included creating an additional physical document that could be retrieved at the library, clinic, or health department if there was an issue with computer or mobile phone access. Other parents described issues related to reading words and visuals on the small mobile screens. Parents recommended increasing font size and white space and creating a complementary paper document to be handed over along with *HPVVaxFacts*. The last concern was the potential for the information to be viewed as “one-sided” or from the provider perspective only.

A few of the providers noted potential disruption of clinic workflow from intervention implementation. In addition, delivering the intervention before the clinic visit could cause a parent to delay or cancel an upcoming wellness or sick visit. It could also create new concerns about the HPV vaccine or about vaccines in general. Another concern was that many parents do not schedule appointments far in advance. Therefore, some parents may not receive the information until shortly before or during the visit. As a result, clinics should map out to whom and how this intervention should be delivered. Language is another barrier for parents who do not speak English or for those for whom English is a second language. The providers also stated that a mobile-based web page limits accessibility*.* Furthermore, the current flux in patient numbers at clinics owing to the COVID-19 pandemic was identified as a barrier. Ensuring confidentiality and privacy of the patient was also a concern. Finally, a provider perceived that many vaccine-hesitant parents would not use the intervention.

### Phase 6: Expert Review of Message Content

Two HPV vaccine experts perceived that the messaging was authoritative. Text was added, rearranged, or removed to increase clarity of a message or if it was considered not essential by the experts; for example, text was removed and added to increase understanding of the importance of vaccine-induced immunity compared with natural immunity. In addition, it was suggested to consistently use either *vaccine immunity* or *vaccine-induced immunity*, and we chose the term *vaccine immunity*. For images, 1 suggestion was to replace the picture for the parental concern related to genital warts to better complement the message content.

### Phase 7: Intervention Modifications and Finalization

#### Overview

We iteratively adapted the tailored health communication intervention *HPVVaxFacts* throughout or after each step based on the feedback and data. Modifications were categorized into the following groups: (1) message content and imagery, (2) intervention design, and (3) delivery. The suggestions and changes made at each phase are presented in Table 2 and briefly summarized in the sections that follow.

**Table 2:** Summary of suggestions and changes at each phase of HPVVaxFacts development.

Phase	Title	Suggestions	Changes
2	MVTCP^a^ CAB^b^ Review	Modify literacy level Make language understandable to lay persons Diversity in images regarding sexual orientation, sex, and race	Lowered literacy level to grade 7 using simple terms Substituted scientific words or added definitions Updated images to be more racially diverse and more male oriented
3	Parental Advisory Panel Review	Need diversity in images (ie, in terms of race, age, and sex) Add images of physicians reflective of all races in white coats Add videos to complement content Add adolescents’ corner (images, messages, and testimonials for adolescents)	Updated more images to reflect diversity in age, race, and sex for children, parents, and physicians Developed videos from providers belonging to different racial groups Added videos that reinforced content of each concern Adolescents’ corner added with images and messages
4	CEHC^c^ Content Review	Reorder or remove information Remove manipulative or contradictory language	Removed problematic language (eg, “as a good parent”) Revised misleading statements (eg, how HPV^d^ is spread) Reordered and revised content
**5**			
	Semistructured Interviews With Parents	Modify content to increase clarity Add images for diversity Add sources for content Add more research-based studies Tailor content to adolescents’ medical condition Introduce research team Increase font size and white space Add guidance on navigation	Updated 3 infographics to increase clarity on content (eg, impact of HPV vaccine on cancer rates) Updated links to go to visuals (eg, genital warts) Updated 2 images to reflect sex and diversity Substituted, added, or removed content from each concern (eg, edited language on rationale for age recommendation) Identified and added sources for content to increase credibility Added About Us tab of research team members with pictures and bio Shortened sentences, added white space, and increased font size Updated instructions on web page
	Semistructured Interviews With Providers	Give providers information on content to prepare for discussion with parents	Developed 1-page summary for providers on vaccine concerns Offered demonstration for providers
6	Expert Review	Edit content to improve accuracy and clarity Change 1 image to be more content appropriate	Edited content for all concerns (eg, updated explanation of natural immunity vs vaccine immunity) We did not change the picture for genital wart content because parents wanted the option to click a link that would lead to a picture of genital warts

^a^MVTCP: Meharry-Vanderbilt–Tennessee State University Cancer Partnership.

^b^CAB: community advisory board.

^c^CEHC: Center for Effective Health Communication.

^d^HPV: human papillomavirus.

#### Message Content and Imagery

The final message library addressed 9 concerns related to the HPV vaccine: safety, recommended age, effectiveness to prevent genital warts and cancers, too many vaccines for adolescents, preference for natural immunity over vaccine immunity, need to prevent cancer, need to prevent genital warts, and the perceptions that vaccine receipt will encourage sexual activity and cause serious health problems to the child. Refer to Textbox 1 for an example message for a tailored variable before and after the iterative message development process.

**Example message for tailoring a variable before and after the iterative development process.**

- Tailoring variable: natural immunity vs vaccine immunity
- Quiz item: I wonder if natural immunity against HPV is better than getting the HPV vaccine
- Initial message before the development process
  - Like you, some parents question whether natural immunity is better than HPV vaccination to protect their child’s health.
  - Although natural immunity from HPV provides immunity like the HPV vaccine does, the risk with HPV infection is much higher. With natural infections, a child might develop complications such as genital warts and cancer. On the other hand, if your child is exposed to a disease like HPV after being vaccinated, he or she would already be armed and able to fight it off.
  - This means your child does not have to get sick from HPV first to develop protective antibodies.
  - All children need their HPV shots to prevent cancer and genital warts.
  - HPV is a very common virus with 14 million affected yearly including teens.
  - Almost all people (4 out of 5) will be affected at some point in their lifetime.
  - 1 person gets HPV every 20 minutes of every day.
  - Over 45,000 cases of HPV cancers a year could be prevented with HPV vaccination.
  - HPV infections can cause:
    - cancers of the cervix, vagina, and vulva in women
    - cancers of the penis in men; and
    - cancers of the anus and back of the throat, including the base of the tongue and tonsils in both men and women
    - genital warts in both men and women.
- HPV-linked throat cancers are highest in men with a 225% increase in cases.
- Final message after the development process
  - Natural immunity happens when your child gets the HPV infection and makes antibodies to fight the infection. The problem with this type of immunity is the danger that getting the infection can lead to genital warts and/or cancer later in life.
  - Immunity through vaccination is when the HPV vaccine causes the body to develop protective antibodies before coming into contact with the virus. This means your child’s body will be armed with protective antibodies that stop the most common types of HPV from infecting them and causing cancer and/or genital warts.
  - Vaccination is much safer than natural immunity. For natural immunity, your child will catch HPV infection which will place them at risk of genital warts and cancer later in life while vaccination will protect your child from infection with the most common HPV types.
  - Vaccination produces antibodies and infection fighting cells called T cells that provide stronger and longer protection against HPV compared to natural infection. There is no evidence of protection decreasing from the vaccine over time.
  - It is important to remember that there are many different types of HPV. Infection provides immunity against one specific type of HPV, while the HPV vaccine provides protection against many different HPV types that cause cancer and genital warts.

#### Intervention Design

**Figure 2:**
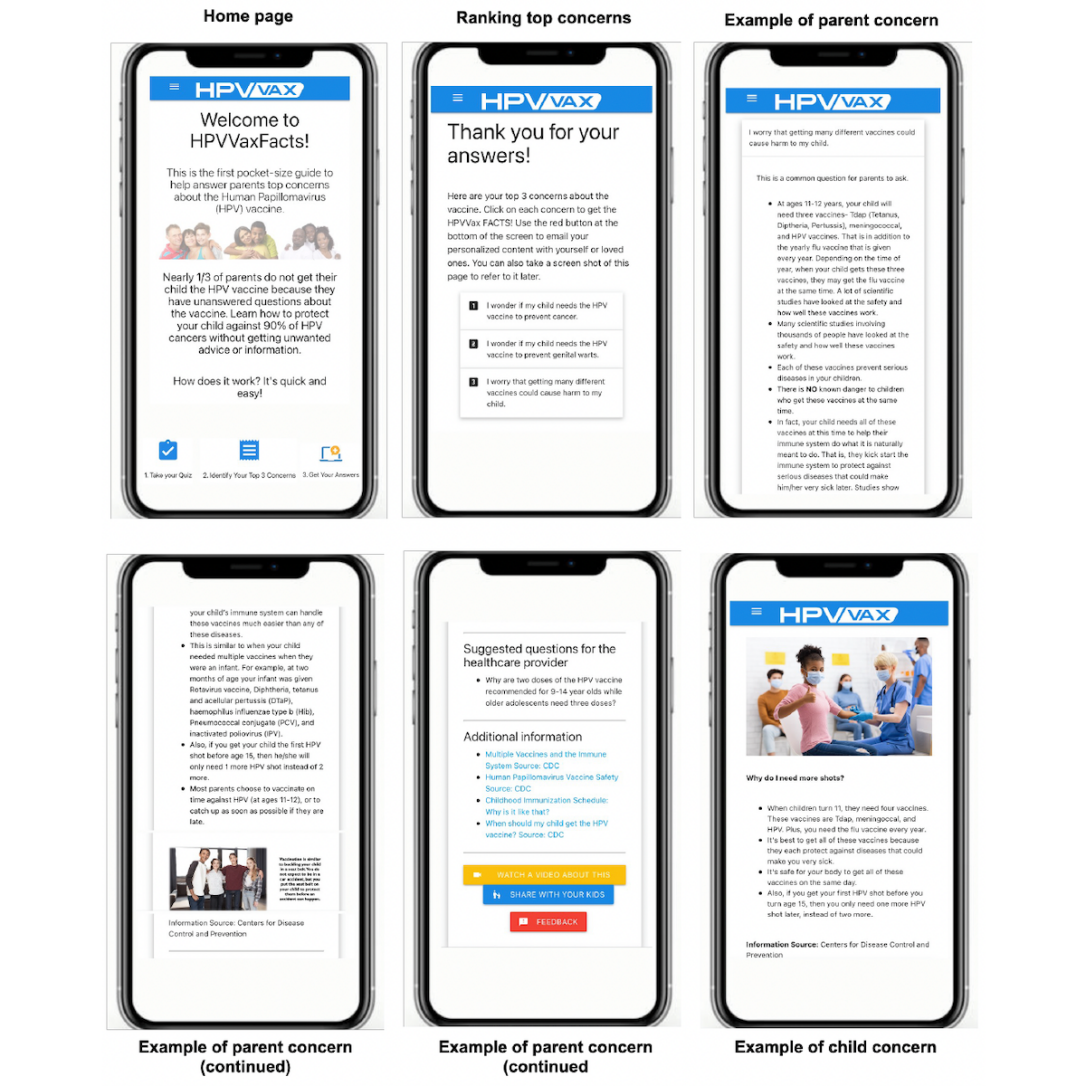
Screenshots of the HPVVaxFacts intervention.

The images on the web page were edited throughout to increase diversity while promoting inclusion. An *About Us* tab was added to introduce the project and team members to the parents. Several features were added based on feedback, including (1) allowing parents to manually rerank their top 3 concerns, if desired; (2) an *adolescents’ corner* on each concern for parents to optionally share with their child, if desired; and (3) option for parents to send the information on their top concerns to themselves via email or text or print it out for future reference. Refer to Figure 2 for screenshots of the intervention.

#### Intervention Delivery

The intervention was programmed as a web-based application that can be viewed on mobile phones as well as PCs. In addition, programming was adapted to ensure that this intervention could be viewed on different web browsers, including Safari, Google Chrome, and Mozilla Firefox.

## Discussion

### Principal Findings

We provided a detailed description and key findings from the multiphase stakeholder-engaged process used to develop a tailored health communication intervention, *HPVVaxFacts*, for vaccine-hesitant parents with adolescent children aged 11 to 18 years. This mobile phone–based web application was developed with the idea of understanding the intersection of theory, tailoring, and community engagement as it relates to the uptake of HPV vaccination among children with HPV vaccine–hesitant parents. The iterative feedback was interwoven systematically into the intervention design and content [56]. The use of these approaches prompted several changes in the design and content of the mobile phone–based web application, which improved cultural appropriateness (eg, diversity and inclusiveness in content, images, and videos) [57]; relevance; and comprehension, which is a limitation cited in past studies [33,58]. Almost all parents and providers found this intervention acceptable, including the delivery of the intervention through the medical home before clinic visits. Parents and providers indicated that this intervention could be beneficial and effective in addressing concerns around HPV vaccination and facilitate parent-child communication. Furthermore, after reviewing the intervention, almost all of the previously vaccine-hesitant parents (29/31, 94%) stated that they intended to have their child vaccinated against HPV.

### Comparison With Prior Work

This study contributes critical information to the small but growing body of research conducted on mHealth interventions for HPV vaccination [29]. Although previous studies have shown promising results suggesting a positive impact on parental decision-making for HPV vaccination [32,33,59], this study fills important gaps and overcomes limitations of existing interventions. First, we used an individually tailored intervention approach to address the varying parental concerns about HPV vaccination while adapting to the context, place, and time [60]. Vaccine safety concerns, the lack of knowledge of the role of HPV in cancer pathogenesis, concerns of encouraging sexual activity after HPV vaccination, and both misinformation and disinformation related to HPV infection and vaccine are common reasons that parents report for vaccine hesitancy and are addressed in the *HPVVaxFacts* intervention [60-63]. *HPVVaxFacts* allows parents to identify their top concerns about the vaccine to obtain factual information tailored to their concerns, a unique feature that does not exist in the handful of other mHealth interventions related to HPV vaccine. In previous studies, Kim et al [32] developed *Vax4HPV* to offer tailored information by vaccine status, and Becker et al [59] created *HPVCancerFree* to offer tailored reminders. Second, similar to Woodall et al [33], we offer a section in *HPVVaxFacts* for adolescents. By contrast, based on parental input, we gave parental autonomy over choosing whether to allow adolescents to view information and which concerns they deemed appropriate. These findings demonstrate that parents differ in their degree of readiness to introduce and discuss HPV and the vaccination. Third and last, this mobile phone–based web application can be used by parents of adolescents of all ages. Past mHealth studies have primarily targeted HPV vaccination in younger adolescents [32,33,58], which limits the impact of these tools on vaccine-hesitant parents who delay vaccination to older adolescents.

The acceptability of mHealth intervention use in clinic settings by both parents and providers is critical to increase the likelihood of use and effectiveness. Both parents and providers indicated that the medical home was the preferred setting for intervention delivery, which concurs with previous research findings that the provider is considered a trusted information source [64]. However, parents have often cited weak recommendations and poor communication from providers surrounding HPV vaccination and the need for providers to offer supplemental materials (eg, web pages) [22,23]. In addition, provider *burnout* or discomfort in convincing parents to get their child the HPV vaccine negatively affects parental decision-making [15,65,66]. Parents and providers confirmed the acceptability of previsit delivery of this intervention to help address parental concerns and increase vaccine acceptability; address most, if not all, provider communication barriers; facilitate parent-child communication; and ultimately increase vaccine uptake among adolescents with vaccine-hesitant parents. A previous study found that previsit HPV vaccine information was a preference for parents [22]; yet, to our knowledge, this strategy has not been applied with an mHealth intervention. Of interest, most parents wanted information 2 weeks to a month before the clinic visit for reviewing it compared with providers stating that the information should be delivered in the waiting room. This finding suggests that parents need more time for decision-making and potential parent-child communication. Previsit information and recommendations starting as early as when the children are aged 9 years [67] could allow more time for vaccine-hesitant parents to obtain answers to their questions and think about their decision.

### Limitations

Early during the COVID-19 pandemic, clinics did not have the capacity to conduct research activities. Therefore, we had to switch to recruiting parents from ResearchMatch, a voluntary research registry, which could be biased toward people who have an interest in research participation. Another potential limitation is the pushback against COVID-19 vaccination and increased general vaccine hesitancy during the pandemic, which may have caused and increased hesitancy among these parents [68]. Furthermore, the child’s HPV vaccination status was by self-report without medical record confirmation. Parents were also recruited and interviewed via a web-based platform, which could have limited perspectives from those who lack digital access. This intervention has an *adolescents’ corner* component based on recommendations from parents, but we did not collect data directly from adolescents to include their input in the development of this component, given that the parents are the primary target audience. In the subsequent pilot study, we will obtain feedback from adolescents.

Parents were recruited from only the southeastern region of the United States. Therefore, it is possible that this region’s local context of vaccine hesitancy may differ from that of other regions. However, the southern region is an important area to focus on because the states in this region generally have lower rates of HPV vaccination and higher levels of vaccine hesitancy than other regions in the United States [69]. This intervention has only been developed for English-speaking parents, although we plan to add additional languages in the future. For providers, recruitment was conducted via snowball sampling, which could lead to sampling bias. For both parents and providers, the sample was small and purposive. Although this is common in qualitative research, this type of sample is not designed to produce results that can be generalized to a specific population. Future work should quantitatively explore views in these populations using probability samples for generalizable results.

### Next Steps

We are completing pretesting to optimize the intervention study protocol and implementation procedures (eg, the timing of intervention delivery before the clinic visit and adoption into the clinic setting). Next, we will conduct a pilot randomized controlled trial to establish the feasibility of the intervention protocol and obtain preliminary data for a full-scale randomized controlled trial.

### Conclusions

Strategies are needed to promote HPV vaccination because parental hesitancy continues to rise and threaten adolescent prevention against HPV-related cancers. We developed a theory-driven tailored health communication intervention using community-engaged research processes. The iterative development of our intervention with the input of the primary target audience, vaccine-hesitant parents, was critical and necessary, given the unique needs of this population, which made it more challenging in terms of changing attitudes and behavior. The input of providers and vaccine experts provided added value and enhanced acceptability. Our findings and the resulting intervention contribute to advancing the science around addressing parental vaccine hesitancy for HPV vaccine or other vaccines. Future research could adapt this intervention for use in other settings such as schools, health departments, and pharmacies. In addition, the systematic stakeholder-engaged development process documented here may be replicated in future research to design other mHealth interventions.

## Abbreviations

CAB: community advisory board
CEHC: Center for Effective Health Communication
HBM: health belief model
HPV: human papillomavirus
mHealth: mobile health
MVTCP: Meharry-Vanderbilt–Tennessee State University Cancer Partnership
REDCap: Research Electronic Data Capture
TRA: theory of reasoned action

## Data Availability

The supporting data cannot be made openly available because of the confidentiality agreements.
