# Correction: Development of a Tailored Mobile Phone–Based Intervention to Facilitate Parent-Child Communication and Build Human Papillomavirus Vaccine Confidence: Formative Qualitative Study

**DOI:** 10.2196/48412

**Published:** 2023-05-04

**Authors:** Jennifer Cunningham-Erves, Consuelo H Wilkins, Amanda F Dempsey, Jessica L Jones, Chris Thompson, Kathryn Edwards, Megan Davis, Lindsay S Mayberry, Douglas Landsittal, Pamela C Hull

**Affiliations:** 1 Department of Internal Medicine Meharry Medical College Nashville, TN United States; 2 Vanderbilt University Medical Center Nashville, TN United States; 3 Merck Denver, CO United States; 4 Meharry Vanderbilt Alliance Nashville, TN United States; 5 233 Analytics Nashville, TN United States; 6 Indiana University Bloomington, IN United States; 7 University of Kentucky Lexington, KY United States

In “Development of a Tailored Mobile Phone–Based Intervention to Facilitate Parent-Child Communication and Build Human Papillomavirus Vaccine Confidence: Formative Qualitative Study” (JMIR Form Res 2023;7:e43041), two errors were noted.

- The name of one author, Jennifer Cunningham-Erves, was erroneously replaced with Jennifer Erves and was originally published as follows:

Jennifer Erves^1^, PhD, MPH, MAEd, MS; Consuelo H Wilkins^2^, MD, MSCI; Amanda F Dempsey^3^, MD, PhD; Jessica L Jones^4^, MSc; Chris Thompson^5^, BSc; Kathryn Edwards^2^, MD; Megan Davis^2^, BSc; Lindsay S Mayberry^2^, PhD; Douglas Landsittal^6^, PhD; Pamela C Hull^7^, PhD

In addition to the author's name, the order of degrees listed for the authors have been changed to appear from lowest to the highest per journal guidelines and now appears as follows:

Jennifer Cunningham-Erves^1^, MSc, MA, MPH, PhD; Consuelo H Wilkins^2^, MSCI, MD; Amanda F Dempsey^3^, MD, PhD; Jessica L Jones^4^, MSc; Chris Thompson^5^, BSc; Kathryn Edwards^2^, MD; Megan Davis^2^, BSc; Lindsay S Mayberry^2^, PhD; Douglas Landsittal^6^, PhD; Pamela C Hull^7^, PhD

- The citation information of the article in the original article was published as follows:

Erves J, Wilkins C, Dempsey A, Jones J, Thompson C, Edwards K, Davis M, Mayberry L, Landsittal D, Hull PDevelopment of a Tailored Mobile Phone–Based Intervention to Facilitate Parent-Child Communication and Build Human Papillomavirus Vaccine Confidence: Formative Qualitative Study JMIR Form Res 2023;7:e43041 URL: https://formative.jmir.org/2023/1/e43041 DOI: 10.2196/43041

The author's name has been corrected in this section and now appears as follows:

Cunningham-Erves J, Wilkins C, Dempsey A, Jones J, Thompson C, Edwards K, Davis M, Mayberry L, Landsittal D, Hull PDevelopment of a Tailored Mobile Phone–Based Intervention to Facilitate Parent-Child Communication and Build Human Papillomavirus Vaccine Confidence: Formative Qualitative Study JMIR Form Res 2023;7:e43041 URL: https://formative.jmir.org/2023/1/e43041 DOI: 10.2196/43041

The correction will appear in the online version of the paper on the JMIR Publications website on May 4, 2023 together with the publication of this correction notice. Because this was made after submission to PubMed, PubMed Central, and other full-text repositories, the corrected article has also been resubmitted to those repositories.

